# Noncoding RNA Profiles in Tobacco- and Alcohol-Associated Diseases

**DOI:** 10.3390/genes8010006

**Published:** 2016-12-23

**Authors:** Nayra Soares do Amaral, Natalia Cruz e Melo, Beatriz de Melo Maia, Rafael Malagoli Rocha

**Affiliations:** 1 Molecular Morphology Laboratory, AC Camargo Cancer Center, São Paulo 01508-010, Brazil; namaral@cipe.accamargo.org.br (N.S.d.A.); beatriz.melomaia@gmail.com (B.d.M.M.); 2 Molecular Gynecology Laboratory, Gynecologic Department, Federal University of São Paulo, São Paulo, Brazil, postcode: 04039-032; natallimel@hotmail.com

**Keywords:** noncoding RNAs, microRNAs, long noncoding RNAs, tobacco, alcohol, cancer, chronic obstructive pulmonary disease, cardiovascular diseases

## Abstract

Tobacco and alcohol are the leading environmental risk factors in the development of human diseases, such as cancer, cardiovascular disease, and liver injury. Despite the copious amount of research on this topic, by 2030, 8.3 million deaths are projected to occur worldwide due to tobacco use. The expression of noncoding RNAs, primarily microRNAs (miRNAs) and long noncoding RNAs (lncRNAs), is modulated by tobacco and alcohol consumption. Drinking alcohol and smoking cigarettes can modulate the expression of miRNAs and lncRNAs through various signaling pathways, such as apoptosis, angiogenesis, and inflammatory pathways—primarily interleukin 6 (*IL-6*)/signal transducer and activator of transcription 3 (*STAT3*), which seems to play a major role in the development of diseases associated with these risk factors. Since they may be predictive and prognostic biomarkers, they can be used both as predictors of the response to therapy and as a targeted therapy. Further, circulating miRNAs might be valuable noninvasive tools that can be used to examine diseases that are related to the use of tobacco and alcohol. This review discusses the function of noncoding RNAs in cancer and other human tobacco- and alcohol-associated diseases.

## 1. Introduction

Tobacco consumption and alcohol abuse are the most prevalent environmental risk factors for the development of diseases worldwide. According to the Global Information System on Alcohol and Health (GISAH), harmful alcohol use is a significant element in over 60 types of diseases and results in the deaths of 3.3 million persons annually [1]. The main diseases that are associated with alcohol abuse are cancers and cardiovascular diseases [2].

Tobacco use is the cause of mortality for approximately 6 million persons each year [1]. Although several tobacco consumption control programs have been initiated globally, the decline in the prevalence of smoking in the US has been less pronounced than in previous decades [3]. According to projections, 8.3 million deaths are expected to be associated with tobacco use worldwide in 2030—one-third of which will be caused by cancers, followed by cardiovascular disease and chronic respiratory diseases [4].

Despite the well-established knowledge on alcohol and tobacco abuse as risk factors for various diseases, their rates of consumption in many countries remain high [5], necessitating a greater understanding of the mechanisms in diseases that are linked to tobacco and alcohol abuse. Environmental factors can influence epigenetic events, such as DNA methylation, histone modifications, and the expression of noncoding RNAs (ncRNAs) [6,7]. Notably, several studies have demonstrated the function of noncoding RNAs in disease development with regard to smoking and alcohol use [8,9]. These molecules have been shown to be new biomarkers in human diseases, because they can leave specific signatures in biofluids and may contain information about the aggressiveness and response to therapy [10,11,12,13].

Based on the impact of the consumption of alcohol and tobacco on human health, the purpose of this review is to integrate the principal findings on this topic from the past 5 years to increase our understanding of the function of noncoding RNAs involved primarily in cancer and in other human diseases associated with tobacco use and alcoholism and highlight their importance as a new diagnostic and therapeutic approach.

## 2. Noncoding RNAs

For many years, it was believed that the genetic information in DNA was transcribed into RNA and subsequently translated into protein (the central dogma of biology). However, noncoding RNAs that can regulate target genes at the posttranscriptional level have been extensively studied [14]. ncRNAs are RNA transcripts that do not encode for a protein and are classified by size as small ncRNAs (sncRNAs), which are shorter than 200 bp, and long ncRNAs (lncRNAs)—those that are longer than 200 bp [15]. microRNAs (miRNAs), which are ≈22 nucleotides (nt) in length, are the most well-studied ncRNAs and have been linked to the consumption of tobacco and alcohol with regard to the development of tumors and other diseases.

### 2.1. miRNAs

It is estimated that more than 60% of the mammalian genome is regulated by microRNAs [16]. A single miRNA is able to simultaneously regulate hundreds of mRNA sequences, highlighting the vast regulatory potential of these molecules. At the same time, a single mRNA can be regulated by several miRNAs [17].

miRNAs are known to regulate gene expression by binding to the 3′-untranslated region (3′-UTR) of messenger RNAs (mRNAs), to promoter regions [18], proteins [19], DNA [20] and Toll-like receptors (*TLR*) [21]. Direct regulation of the epigenetic machinery [22] has also been extensively demonstrated. miRNAs are not only able to downregulate, but also upregulate the expression of target genes [23].

A total of 1881 precursors and 2588 mature human miRNA sequences have been described [24]. miRNAs are involved in signaling pathways during the development of some diseases, primarily cancer [25,26].

The expression of mature miRNAs is mediated by several enzymatic complexes that regulate their biogenesis. The first step of miRNA biogenesis (the canonical pathway) is the transcription of a miRNA gene by RNA polymerase II or RNA polymerase III into primary transcripts (pri-miRNA) that contain a stem loop structure [27,28]. Next, two enzymes, DROSHA and DiGeorge critical region 8 (DGCR8), recognize the pri-miRNA stem loop, which is cropped into a precursor miRNA (pre-miRNA). Pre-miRNAs have approximately 70 nucleotides and are transported from the nucleus to the cytoplasm by exportin-5 (XPO5) and are later processed into mature miRNAs (21 nt) [25]. In the cytoplasm, pre-miRNA* duplexes are processed by Dicer and other proteins, such as TAR RNA-binding protein (TRBP) and kinase R-activating protein (PACT). The miRNA duplex comprises two strands, one that is stabilized by Argonaute (AGO) protein and subsequently incorporated into an RNA-induced silencing complex and another that is usually degraded. The mature miRNA is integrated into a miRNA-mediated silencing complex (miRISC) and suppresses the expression of target genes, which are degraded, destabilized, or translationally inhibited [29].

Several studies have shown that changes in miRNA biogenesis, methylation, polymorphisms, mutations in miRNAs, and altered microenvironments, such as in hypoxia, contribute to dysregulated expression of miRNAs and accelerate the development of diseases [30,31,32,33]. Tobacco and alcohol induce changes in genes that can be related to miRNA maturation after cell exposure to these risk factors; there has been a reduction in the levels of mature miRNA and the expression and activity of Dicer [31,34], one of the main enzymes involved in miRNA biogenesis.

### 2.2. lncRNAs

lncRNAs are able to interact with DNA, RNAs, and proteins [35]. For a long time, lncRNAs were considered to be non-functional, but, currently, these molecules appear to be the main regulators of transcription and translation [36] and have many functions, such as activation and repression of transcription, RNA enhancer, scaffolding protein for chromatin remodeling complexes, regulation of splicing of RNA, and sequestration of miRNA [35]. LncRNAs are transcribed by RNA polymerase II, and have a 5′-cap and a 3′-poly-A tail that can also be alternatively spliced [37]. Compared to mRNAs, lncRNAs frequently reside in the nucleus, are more tissue-specific, and have poorer interspecies sequence conservation [38]. The expression of lncRNAs in many tissues is important for homeostatic processes, including differentiation, organogenesis [39], and the immune response [40].

However, the dysregulation of lncRNAs is associated with the development of neurodegenerative [41], cardiovascular [42], and metabolic diseases [43] and cancer [44], including diseases associated with risk factors, such as tobacco and alcohol [44,45]. In this context, lncRNAs can be the key to understanding the development of normal and pathological biological processes. In this review, we report studies involving lncRNAs in diseases related to the use of tobacco and alcohol and their application as biomarkers and therapeutic targets.

## 3. Diseases and Smoking: An Introduction

The first report on the consequences of tobacco consumption on health was published in 1950 by Doll and Hill [46], who identified an association between cigarettes and lung cancer. Since then, the vast research in this area has expanded the number of tobacco-related diseases [47,48]. Cigarettes have over 7000 harmful chemicals and carcinogens [49,50] that are involved in developing several types of tumors and respiratory and cardiovascular diseases [49,51,52,53].

### 3.1. miRNAs in Tobacco-Associated Diseases

#### 3.1.1. Lung Cancer

Lung cancer is a major disease that is associated with tobacco use; this risk factor is responsible for 75% of all such cases of this disease. Lung cancer is usually diagnosed in the late stages [54,55], and its early detection in smokers can significantly reduce mortality rates [54]. miRNAs are notable targets for this approach and have been exploited in lung cancer. Techniques for analyzing miRNA expression on a large scale have increased our knowledge of the profiles of these molecules in smokers with and without cancer [8,11,56,57,58] and in patients with cancer who have never smoked [59,60,61,62].

The combination of computerized tomography (CT) and the evaluation of miRNA expression reduces false-positive rates and increases the accuracy of early diagnosis in lung cancer [63,64,65]. The upregulation of miR-21 and miR-210 and the downregulation of miR-486-5p correlate with the use of tobacco and with the size of pulmonary nodules. In addition, the alteration of these miRNAs distinguishes patients with malignant pulmonary nodules from those with benign nodules with a sensitivity of 75.00% and a specificity of 84.95% [63].

The analysis of circulating miRNAs in body fluids, such as blood and sputum, is advantageous, because it is a minimally invasive technique that has been exploited in the early diagnosis of lung cancer in smoking [12,13,58,64]. The expression of miRNAs in sputum [64] and serum [12,13,58] refines the diagnosis in smokers with lung cancer compared with nonsmokers with good predictive value. In sputum samples, miR-31 and miR-210 are upregulated. In serum samples, miR-20a, miR-223, miR-21, and miR-145 [12,13] are upregulated and let-7i-3p and miR-154-5p are downregulated, suggesting that these miRNAs are potential biomarkers for early detection of lung cancer in smokers [58].

In addition, immune system components have been examined with regard to miRNAs. The expression of miR-19b-3p and miR-29b-3p in peripheral blood mononucleated cells (PBMCs) is related to the diagnosis of different stages of lung cancer [8].

The oral brush is another minimally invasive tool that detects differentially expressed miRNAs and can be a new clinical method for early diagnosis of lung cancer and for initial screening in smokers. The expression of miR-23a, miR-181c, miR-192, miR-194, miR-208, miR-337-5p, miR-338-3p, miR-502-5p, miR-542-3p, miR-628-5p, and miR-672 is upregulated in the oral mucosa of heavy smokers with lung cancer versus patients without cancer and light smokers [66].

miRNAs also have value in the treatment of smoking-related lung cancer [67,68]. miR-4423 is significantly downregulated in tumors of smokers and nonsmokers compared with normal tissue. Therapy with miR-4423 downregulates genes in the phosphatidylinositol 3-kinase (PIK3CA) and SH2-containg protein (SHC1) signaling pathways, which are fundamental for carcinogenesis, highlighting its tumor suppressive activity and its potential as a therapeutic target [67]. Furthermore, miRNAs have a role in the course of therapy; patients with lung cancer who have undergone surgical treatment show an increase in miR-625 and miR-361-3p, with similar expression levels as those in individuals with benign diseases and healthy persons, emphasizing that these miRNAs might have a protective influence on the development of lung cancer [54].

Further, Balansky et al. (2014) [68] determined the effects of lapatinib, a dual tyrosine kinase inhibitor largely used for breast cancer and other solid tumors, on miRNA expression by microarray in mice. Animals that were exposed to mainstream cigarette smoke (MSC) without lapatinib experienced global downregulation of miRNAs compared with the group that was administered the drug. Among the miRNAs that were downregulated by MSC were miR-92, miR-223, miR-27a, miR-139, miR-181c, miR-181, miR-27a, miR-322, miR-489, miR-511, miR-27a, miR-34b, miR-885, miR-19b, miR-19b, miR-20a, miR-292, miR-322, miR-362, miR-19b, miR-216a, miR-326, miR-341, miR-702, miR-19b, and miR-292, which are involved in the regulation of several genes—for example, Dicer, a key molecule in miRNA biogenesis [68]. Notably, the modulation of miRNAs is dependent on the level of tobacco consumption and can influence the prognosis of patients with lung cancer [11].

#### 3.1.2. Other Cancers

The modulation of miRNAs in response to smoking has also been reported in stomach, esophagus, and oral tumors [69,70,71]. Smokers with gastric cancer experience an upregulation of miR-21 and downregulation of miR-143 in comparison with healthy subjects [70].

Xi and colleagues (2015) [69] elegantly demonstrated that miR-217 is downregulated and that its target gene, kallikrein 7 (*KLK7*), increases in esophageal adenocarcinoma cell lines and esophageal tumors that have been exposed to cigarette smoke condensate (CSC). Further, the suppression of miRNA in the cell model and esophageal tumor samples correlated with hypermethylation of CpG islands in the coding region of miR-217. Notably, the reduction in miR-217 expression and the upregulation of *KLK7* effected greater methylation levels in patients with a history of smoking compared which those who had never smoked [69].

In oral cancer, the expression profile of miRNAs in brush cytology of smokers is more heterogeneous than in nonsmokers with this tumor and healthy individuals. miR-10b-5p, miR-196a-5p, and miR-31-5p are upregulated in tumor tissues regardless of smoking habit, whereas miR-637 is upregulated exclusively in smokers, which may refine the diagnosis for this risk group [71].

#### 3.1.3. Chronic Obstructive Pulmonary Disease

Chronic obstructive pulmonary disease (COPD) is characterized by progressive inflammation of the airways, the chief cause of which is cigarette smoking [72]. The heterogeneity of COPD is one of the reasons for the difficulty in developing effective therapies for it [73], prompting many studies to determine the molecular mechanisms of the disease to identify therapeutic targets. The expression of genes such as transforming growth factor-β (*TGF-β*) and tripeptide Gly-His-Lys (*GHK*), is associated with increased severity of regional emphysema in individual lungs, and these genes mediate several pathways, such as inflammation, extracellular matrix remodeling, and tissue repair [74].

Smoking can potentiate the inflammatory process in COPD, altering the expression of miRNAs and these molecules can support in the diagnosis, because they are differentially expressed between groups of normal individuals compared to groups of smokers with COPD [75,76]. miRNAs interfere with the expression of genes that are involved in progressive inflammation of the airways and lungs—let-7c and miR-125b are lower in currently smoking patients with COPD compared with healthy subjects; however, these miRNAs are not differentially altered between ex-smokers and healthy controls. A decrease in let-7c is associated with increased levels of tumor necrosis factor receptor II (*TNFR-II*). The authors suggest that these miRNAs are associated in the development of COPD during exposure to cigarettes but not in the inflammation of airways during smoking cessation [76]. Ezzie et al. (2012) [77] identified 70 miRNAs that were differentially expressed in lung tissue among smokers with COPD and healthy smokers. miR-223 and miR-1274a were upregulated in subjects with COPD versus smokers without obstruction, and miR-15b was increased in COPD patients compared with smokers without obstruction and was differentially expressed between disease severities. In vitro, exposure to cigarette smoke extract upregulates miR-7 and downregulates *EPAC1*—important genes in the pathogenesis of COPD, including inflammation [76].

In addition, tobacco can participate in the alteration of T cells, macrophages, neutrophils, and the expression of miRNAs that play a key role in the immune response [78]. miR-146b-3p, miR-150, and miR-210 are downregulated in alveolar macrophages of smokers compared with nonsmokers. Further, heavy smokers experience global repression of miRNA versus light smokers; three of these miRNAs are differentially expressed (miR-146b-3p, miR-150, and miR-210), as validated in a second set of alveolar macrophage samples [79]. Another study has shown that alveolar macrophages from chronic active cigarette smokers express fewer mature miRNA transcripts, in part due to sumoylation of Dicer [34]. In addition, miR-199a-5p is downregulated in regulatory T cells (Tregs) in patients with COPD versus healthy smokers. Also, miR-199a-5p modulates the response of Tregs through the TGF-β pathway [78].

Further, normal lung fibroblasts that are exposed to cigarette have lower RelB levels when miR-146a is upregulated; this upregulation is significant in COPD lung tissue compared with normal lung [80]. Notably, miR-146a is a potential therapeutic target for COPD, and the rs2910164 polymorphism in it is associated with a decrease in miR-146a levels and a rise in cyclooxygenase 2 (*COX2*) in smokers with COPD [81].

Circulating miRNAs have also been examined as a clinical tool for COPD patients [53,81,82,83]. Shi and colleagues (2015) [83] first identified 11 differentially expressed miRNAs in lung tissues from smokers with COPD, non-COPD smokers, and healthy nonsmoking controls. Five of these miRNAs (miR-181a, miR-203, miR-338, miR-1, and miR-199a) were evaluated in the blood of the three groups, and miR-203 levels were higher in the blood of smokers and COPD patients compared with nonsmoking controls. It has been suggested that miR-203 contributes to the development of COPD through the repression of nuclear factor κ-light-chain-enhancer of activated B cells (NF-κB) signaling by targeting *TAK1* and *PI3KCA* and that this miRNA can be used as a new biomarker for COPD diagnosis. Plasma levels of miR-106b decreased progressively after COPD patients stopped smoking, suggesting that this miRNA is an important clinical marker in plasma that is indicative of COPD [82]. Further, in the plasma of patients with COPD, miR-4455 and miR-4785 were differentially expressed between current smokers and never-smokers [81].

Thus, miRNAs govern vital inflammatory responses, regulating pro-inflammatory cytokines in patients with COPD, rendering them potentially valuable therapeutic and diagnostic tools [72].

#### 3.1.4. Cardiovascular Diseases

Cardiovascular diseases are the leading cause of death in the world; more than 17 million people die each year of cardiovascular diseases, for which cigarette use is one of the main risk factors [84]. Little is known about the changes of miRNAs in the development of these diseases after exposure to smoke or alcohol consumption, but in vitro findings and clinical observations have confirmed that the use of cigarettes spurs the development of cardiovascular diseases [51,52]. Exposure of cardiomyocytes to nicotine induces fibrosis; reduces cell viability and miR-133 levels; and increases membrane potential, DNA degradation, and the expression of caspase 9, a target of miR-133 [51]. In abdominal aortic aneurysms, miR-21 is upregulated in response to nicotine and pathological progression. miR-21 is a notable therapeutic target for cardiovascular disease, because its inhibition in mice inhibits cell proliferation and increases apoptosis in the aortic wall, protecting against expansion of the aneurysm and the effects of nicotine [52].

## 4. Alcohol Abuse and Alcohol-Related Diseases

Excessive alcohol consumption is an important health problem, because it causes over 200 diseases, such as cancer, obesity, and liver and cardiovascular diseases [1,2]. Alcohol abuse is responsible for a significant percentage of mortality from these diseases [1]. Although alcohol and acetaldehyde have relatively low carcinogenicity, the alcohol content is high in alcoholic beverages [85], making alcohol abuse a major risk factor for the development of liver tumors, cancers of the head and neck, and breast tumors [2].

### 4.1. miRNAs in Alcohol-Associated Diseases

#### 4.1.1. Liver Cancer

Liver cancer is one of the most common malignant tumors globally and has the third highest mortality rate, with approximately 700,000 new cases annually worldwide [86]. The main risk factors for hepatocellular cancer (HCC) include alcohol consumption, the presence of cirrhosis, hepatitis B/hepatitis C virus (HBV/HCV) infection, nonalcoholic steatosis, and hepatitis [87,88,89]. Liver cancer generally has a late diagnosis and poor prognosis and is resistant to therapy [90].

Several studies have shown that ethanol modulates microRNA expression in the liver [91,92,93,94], but little is known about the relationship between alcohol consumption and miRNA expression in the development of liver cancer. Nevertheless, alcohol alters the levels of miRNAs that regulate important cellular processes in hepatic tumorigenesis [9,30,91].

Meng and colleagues (2012) [30] reported that the positive regulation of miR-34a after treatment of liver cancer and normal cell lines with alcohol is associated with the hypomethylation of the promoter region of this miRNA. By microarray analysis, miR-34a was differentially expressed in the hepatic tissue of rats after administration of ethanol compared with rats in the control group. In addition, miR-34a has significant function in modulating genes that mediate hepatic fibrosis during cell remodeling, with caspase 2 and sirtuin 1 (*SIRT1*) as direct targets and matrix metalloproteases 1 and 2 (*MMP1* and *MMP2*) as indirect targets [30]. In alcoholic liver tissue, the expression of miR-200 is impaired, and its target, *SPRR2a*, is upregulated. In vitro, transfection of *SPRR2a* into cholangiocarcinoma cells downregulates miR-200 and increases mesenchymal-epithelial transition (MET) genes, such as *DESMOPLAKIN*, *E-CADHERIN*, *CLAUDIN-7*, *LAMININ SUBUNIT BETA-3*, *MUCIN-1*, and *ZEB1* [91]. Francis et al. (2014) [9] observed that normal liver and liver cancer cells that have been treated with ethanol upregulate miR-21—to a greater extent in the latter. In addition, cells that have been transfected with miR-21 showed greater transformation and survival.

The consumption of alcohol might be associated with mechanisms that regulate the expression of miRNAs in liver cancer, such as the modulation of genes that participate in the biogenesis of miRNAs and polymorphisms in miRNA-coding regions [31,32,95]. The expression of seven genes (*DGCR8*, *P68*, *P72*, *DICER*, *AGO3*, *AGO4*, and *PIWIL4*) that mediate the biogenesis of miRNAs declined in liver cancer samples, especially in nonviral tumors, compared with normal liver. Downregulation of these genes correlated with a worse prognosis and the presence of risk factors, such as smoking and alcohol consumption. Also, the promoter regions of *P72*, *AGO4*, and *DGCR8* were methylated in HCC cell lines [31].

Single-nucleotide polymorphisms in miRNAs can alter their expression and their binding to target mRNA sequences; thus, this mechanism might increase one’s susceptibility to tumors [32]. Individuals who have the CT or CC rs3746444 polymorphism in miR-499 might have significantly greater susceptibility to liver cancer. The combined effects of polymorphisms and environmental risk factors, such as smoking and alcohol consumption, have been reported [32].

These data demonstrate that the involvement of alcohol in miRNA modulation in hepatic tumors should be studied further. It is important to increase our knowledge about liver cancer with regard to an early diagnosis and therapeutic resistance.

#### 4.1.2. Other Cancers

Alcohol is a risk factor for head and neck tumors (HNSC), and as shown by RNAseq analysis of these tumors, individuals who consume alcohol have disparate miRNA profiles and lower survival rates compared with those who do not. miR-30a-5p, miR-934, miR-3164, and miR-3178 are upregulated in oral tumors from those who consume alcohol, in oral epithelial tumor cell lines, and normal cells on exposure to acetaldehyde. Cells that are transfected with miR-30a-5p and miR-934 upregulate *BCL2*, which mediates processes such as proliferation and invasion. Notably, cells in which these miRNAs have been knocked down become sensitive to cisplatin and show less proliferation and invasiveness [96].

In oral cancer, miR-34a, miR-99A, miR-143, and miR-380-5p are negatively regulated in patients with cancer compared with healthy subjects. Further, increased expression of miR-34a is associated with alcohol consumption and histological grade, this miRNA had targeted *MDM4*, a negative regulator of *TP53*, suggesting an indirect mechanism of suppression of p53 in oral cancer [97]. In premalignant lesions of the larynx, miR-10a levels are reduced and miR-34c increases gradually by severity of the dysplasia, wherein miR-34c expression levels are higher in patients who consume alcohol [98].

In samples of gastric cancer, there is differential expression of miRNAs in association with alcohol consumption. For example, miR-203, miR-205, and miR-223 are upregulated in patients with gastric cancer who consume alcohol regularly [70].

#### 4.1.3. Other Alcoholic Liver Diseases

Alcoholic liver disease (ALD) is a spectrum of diseases—including steatosis, hepatitis, and alcoholic cirrhosis—that can progress to hepatocellular carcinoma [99].

An analysis of genes and miRNAs was performed in 4 groups: healthy non-drinkers, healthy drinkers without liver disease, and drinkers with hepatitis or cirrhosis. The results revealed that six miRNAs (miR-570, miR-122, miR-34b, miR-29c, miR-922, and miR-185) were able to downregulate 79 target genes involved in various processes, such as the immune response, inflammation, and glutathione metabolism [100].

miRNAs that are associated with extracellular vesicles (EVs) and exosomes can be used as biomarkers in alcoholic hepatitis [93,101,102]. The number of circulating EVs increases, depending on the dose and length of alcohol exposure in mice. A microarray analysis of exosomes in alcohol-treated mice versus controls showed that miR-192, miR-122, and miR-30a differentiate alcohol-induced liver injury in mice and humans with excellent diagnostic accuracy [102]. Exosomes mediate the communication between alcohol-treated hepatocytes and immune cells, and the levels of miR-27a in exosomes and inflammatory cytokines, such as interleukin 10 (*IL-10*) and *TGF-β*, are higher in alcohol-treated mice and patients with alcoholic hepatitis [101].

miRNAs are considered new targets for the diagnosis of and therapeutic interventions for alcoholic fatty liver (AFL) [30,103]. In mice with AFL, miR-199-3p, miR-214, miR-93, miR-146a, miR-191, and let-7b are downregulated and miR-129, miR-490, miR-21, miR-503, miR-183, and miR-185 are upregulated compared with healthy mice [103].

Alcoholic liver disease is characterized by the activation of a cascade of proinflammatory cytokines in the liver [104]. Bala et al. (2012) [93] demonstrated that circulating miR-122 and miR-155 are biomarkers of liver damage and modulators of proinflammatory cytokines in mice. Further, the upregulation of miR-217 and inflammatory cytokines in Kupffer cells of mice on administration of ethanol reduces *SIRT1*, and increases NF-κB expression. Thus, the inhibition of hepatic miR-217 is an interesting approach toward mitigating the inflammatory response in alcoholic steatosis [105].

In alcoholic and viral hepatitis, miR-122 functions in the regulation and replication of HCV and in the expression of host genes, including *CYCLIN G1*. In cells with and without HCV that have been exposed to acute concentrations of alcohol, miR-122 levels rise [92].

The intestinal barrier dysfunction is commonly associated with alcoholic liver disease. Experiments with monolayer cells and mice showed that exposure to alcohol increases miR-212 and hyperpermeability and downregulates zonula occludens-1 protein. Inhibition of miR-212 can reduce this effect; thus, this miRNA can be used as an interesting therapeutic intervention [106].

**Figure 1:**
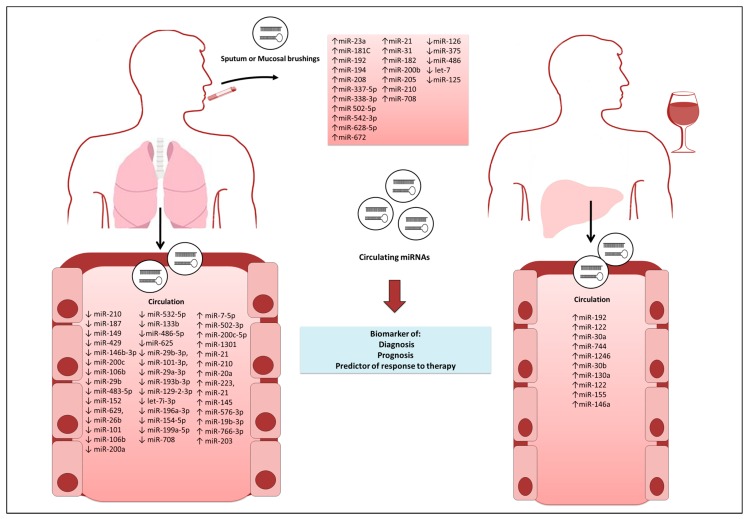
Circulating microRNAs (miRNAs) as early diagnostic biomarkers in tobacco- and alcohol-associated diseases. Circulating miRNAs from blood and sputum or mucosal brushings can be obtained through a minimally invasive technique and have been exploited to assist in the early diagnosis of several diseases.

This review focused on altered miRNAs due to of the use of tobacco and alcohol in various human diseases. It has been shown in diverse studies that exposure to tobacco and alcohol alters the expression of several miRNAs in tumors (Table 1) and other diseases, such as cardiovascular, respiratory, and liver diseases (Table 2), in different samples, such as cells, animals, and humans. One of the main approaches among these studies was to quantify circulating miRNAs that are secreted from cells in body fluids, because they are stable molecules. Circulating microRNAs are advantageous, because they can be obtained through a minimally invasive technique, and they have been exploited to assist in the early diagnosis of lung cancer, COPD, and liver diseases [54,82,102,107] (Figure 1). The use of circulating miRNAs might facilitate the diagnosis of various diseases that are associated with tobacco consumption and alcohol abuse, focusing on the concept that different human diseases leave specific miRNA signatures in biofluids, which contain information about the aggressiveness and response to therapy.

## 5. Long Noncoding RNAs in Tobacco and Alcohol-Associated Diseases

lncRNAs are involved in tobacco and alcohol consumption-associated carcinogenesis [110,111]. Because the findings on lncRNAs and environmental risks are relatively new, we will focus on the function of these molecules in lung cancer and hepatocellular carcinoma—the areas in which there is more relevant information.

Hepatic reduction in the expression of *SIRT1* promotes steatosis, inflammation, and fibrosis in response to ethanol challenge in mice [112]. A highly conserved lncRNA, MALAT1 (metastasis-associated lung adenocarcinoma transcript 1), has been demonstrated to have a strong propensity to interact with *SIRT1*. *MALAT1* increases in liver fibrosis and mediates the degradation or inactivation of SIRT1 through its association with the histone deacetylase *SIRT1* [111]. In addition, the overexpression of lncRNA *MALAT1* can predict the recurrence of hepatocellular carcinoma after liver transplantation [45].

The exposure of cells to cigarette smoke extract (CSE) induces the epithelial-mesenchymal transition (EMT) during transformation, and lncRNAs might be involved in inflammation through the EMT in human bronchial epithelial (HBE) cells. In CSE-treated HBE cells, interleukin 6 (*IL-6*) activates phospho-Signal transducer and activator of transcription 3 (STAT3) and increases the levels of lncRNA mRNA. CSE also upregulates HOX transcript antisense RNA (HOTAIR) and MALAT1. By chromatin immunoprecipitation (ChIP) assay, *STAT3* interacts with the promoter regions of HOTAIR. Further, malignant transformation was reversed when cells were treated with HOTAIR small interfering RNA (siRNA) for 24 h [7]. Similarly, *MALAT1* participates in the CSE-induced EMT in HBE cells and is regulated by miR-217. MALAT1 has been implicated in the EMT through the enhancer of zeste homolog 2 (EZH2) pathway. Downregulation of miR-217 increases *MALAT1* levels to induce *EZH2*/histone H3 lysine 27 trimethylation (*H3K27ME3*), and this pathway modifies the EMT and causes malignant transformation in HBE cells [110].

Recently, lncRNA polymorphisms have been studied. rs2839698 in the lncRNA H19 is associated with a lower response to platinum-based chemotherapy, primarily in nonsmoking females with adenocarcinoma, and rs1899663 in HOTAIR correlates with the response to chemotherapy in male smokers in a dominant model [113].

The two most extensively studied lncRNAs in lung cancer with regard to tobacco use are HOTAIR and MALAT1. However, Thai et al. (2013) [44] have reported an association between smoking, lung cancer, and an lncRNA-1 (*SCAL1*) in vitro and in vivo. *SCAL1* is more highly expressed in smokers compared with nonsmokers, and CSE induces its expression in vitro in airway epithelial cultures. Notably, *SCAL1* is also upregulated in several lung cancer cell lines. *SCAL1* is regulated by nuclear factor (erythroid-derived 2)-like 2 (*NRF2*)—a mechanism that might be involved in oxidative stress in airway epithelial cells [44].

Although many studies have examined the function of noncoding RNAs and environmental factors, our understanding of them remains insufficient [114]. Recent studies have evaluated the activity of lncRNAs in the development of diseases that are associated with tobacco and alcohol abuse, but little is known about the mechanisms behind this progression. However, the data that are being generated are providing insights into how lncRNAs are involved in diseases that are associated with environmental factors and how they can guide the development of new clinical tools.

## 6. Noncoding RNAs Involved in Signaling Pathways

According to the studies addressed in this review, the major miRNAs associated with tobacco and alcohol are miR-21, miR-34a, miR-34c [30,97,98,109], miR-223 [12,70], miR-375, and miR-210 [63,64], which are involved in many signaling pathways, such as proliferation [52], transformation [9,13], inflammation [9,13], angiogenesis [13], apoptosis, and the cell cycle [12,30,70,71,98,109].

Tobacco and alcohol seem to regulate miR-34c and miR-223 through the signaling pathways of apoptosis and the cell cycle [12,30,70]. miR-34c is downregulated in laryngeal premalignant epithelial lesions [98] and lung cancer, associated with the use of tobacco [109], and upregulated in two studies related to alcohol consumption [30,97]. Exposure to alcohol through miR-34a reduces the expression of target pro-apoptotic genes (*CASPASE 2*, *MDM4*) and a microtubule regulator (*SIRT1*) and is involved with the remodeling of the extracellular matrix (*MMP1* and *MMP2*) [30]. The upregulation of miR-223 has been reported in two studies in smoking patients with lung cancer [12] and in gastric cancer after exposure to alcohol [70]. The targets of miR-223 are a tumor suppressor, tumor protein p53-inducible nuclear protein 1 (*TP53INP1*), and a microtubule regulator, stathmin 1 (*STMN1*) [115,116]. Thus, miR-34a and miR-223 are interesting molecules to be studied in conditions associated with the use of tobacco and alcohol for the development of new tools for diagnosis and therapy.

According to our findings, the upregulation of miR-210 is a non-invasive biomarker in serum samples [63] and sputum [64]. The increased expression of miR-210 is also detected in a variety of other tumors and correlates with advanced breast and oral cancers [117,118]. In the literature, miR-210 is associated with tumorigenesis and hypoxia pathways [119,120]. miR-375 was found in two studies. Whereas Mascaux and colleagues (2013) [109] showed that miR-375 is upregulated in current smokers compared with former smokers, Shen and colleagues (2014) [64] reported that it is downregulated between smokers with and without lung cancer. Despite the interesting findings of the direct association of miR 210 and miR-375 with smoking and miR-223 with tobacco and alcohol use, the pathways involved between these miRNAs and the use of these risk factors have not been elucidated.

Of the miRNAs in tobacco- and alcohol-associated diseases that have been discussed in this review, miR-21 is the most frequently reported, having been described in eight studies—five on tobacco use and two on alcohol consumption. miR-21 regulates the expression of genes that mediate apoptosis, proliferation, invasion, and angiogenesis [9,13,52,108]. miR-21 is associated with tobacco use in lung tumors [12,58,63], gastric tumors [70] and abdominal aortic aneurysms [52]. In liver diseases, miR-21 has also been linked to alcohol consumption [9,103]. Thus, evaluating miR-21 expression can be helpful in diagnosing the early stages of lung cancer [12,63]. In gastric tumor samples, elevated miR-21 expression correlates with smoking and low social status [70].

**Figure 2:**
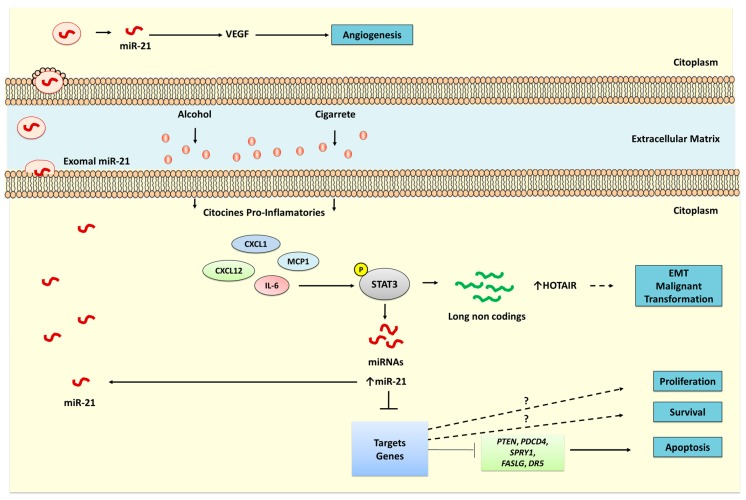
Induction of cellular inflammatory processes by tobacco and alcohol. Tobacco and alcohol induce inflammatory processes primarily through the interleukin 6 (IL-6)/signal transducer and activator of transcription 3 (STAT3) pathway. Phosphorylation of STAT3 by IL-6 governs the expression of miRNAs and long noncoding RNAs (lncRNAs). In aortic artery aneurysms and lung and liver cancers, miR-21 is associated with alterations in the IL-6/STAT3 pathway. Cells chronically exposed to cigarette smoke extract (CSE) show increased expression and secretion of miR-21 in exosomes and greater angiogenesis. CSE also induces the epithelial–mesenchymal transition (EMT) and upregulates the HOX transcript antisense RNA (HOTAIR) lncRNA. This pathway is involved in many processes, thus representing a new approach for identifying therapeutic targets in disorders associated with the use of alcohol and tobacco. miR-21: microRNA-21; VEGF: Vascular endothelial growth factor; MCP1: monocyte chemotactic protein-1; CXCL12: stromal cell–derived factor-; CXCL1: chemokine C-X-C motif ligand 1; IL-6: interleukin-6; STAT3: Signal transducer and activator of transcription 3; *PTEN*: phosphatase and tensin homolog; *PDCD4*: programmed cell death 4; *SPRY1*: sprouty-1; *FASLG*: TNF superfamily, member 6; *DR5*: death receptor 5.

Exposure to tobacco and alcohol modulates the expression of lncRNAs, such as MALAT 1 and HOTAIR, and miRNAs through the IL-6/STAT3 pathway (Figure 2). Liu et al. (2016) [13] and Francis et al. (2014) [9] showed that the regulation of miR-21 in lung and liver cancers is associated with the use of cigarettes and alcohol, respectively, by this pathway. IL-6 phosphorylates and activates STAT3, which in turn activates miR-21 [9,13].

miR-21 is upregulated in the serum of smokers compared with nonsmokers and correlates with high tobacco consumption. In vitro, transformed HBE cells that have been chronically exposed to CSE express and secrete more miR-21 in exosomes and experience increased angiogenesis. In addition, *STAT3* regulates miR-21 levels and the secretion of exosomes in transformed cells. Notably, the supernatant from transformed cells induces angiogenesis through vascular endothelial growth factor (VEGF) in cells that have not been exposed to CSE [13].

Normal human hepatocytes (N-Hep), human hepatic stellate cells (HSCs), and hepatocellular carcinoma cells (HepG2) that are treated with ethanol upregulate miR-21, mainly in tumor cells. Further, normal cells that have been transfected with miR-21 have an increased capacity to transform as well as to survive, downregulating their proapoptotic target genes, *FASLG* (TNF superfamily, member 6) and *DR5* (death receptor 5). Liver tissue that has been exposed to ethanol expresses more phosphorylated STAT3 and miR-21. Silencing IL-6 in normal and hepatocarcinoma cells that have been exposed to ethanol significantly reduces miR-21 expression and phosphorylated STAT3 activity, as shown in animal models [9].

In abdominal aortic aneurysms, mice and cells that have been exposed to nicotine and smoking patients have high levels of miR-21, which correlates with a reduction in tumor suppressors, like *PTEN* and *PDCD4*; upregulation of inflammatory genes, such as *CXCL1*, *CXCL12*, *Il6*, and *MCP1*; and disease severity. In addition, cells that have been transfected with anti-miR-21 downregulate *Ki-67*, a proliferation marker, and express high levels of proapoptotic genes, such as *caspase 3* and *PTEN* [52].

## 7. Final Considerations

Despite the various interventions to discourage tobacco and alcohol consumption, they remain the chief risk factors in the development of many diseases worldwide. The discovery of new molecules that are able to assist in the diagnosis and treatment can reduce the incidence and mortality of these diseases. Many studies have demonstrated that in various experimental models—cell lines, animals, tissues, and human fluids—miRNAs are associated with disease, for which alcohol consumption and smoking are potential risk factors. Several advances have been realized using ncRNAs in translational research; however, limited studies have reported the biological processes in which these molecules and mechanisms are involved.

The most prevalent studies are the associations between lung diseases in smokers and miRNAs. Yet, little is known about the influence of alcohol and tobacco on the expression of ncRNA in cardiovascular diseases.

Exposure to tobacco and alcohol modulates the expression of miRNAs and lncRNAs through the IL-6/STAT3 inflammatory pathway, and this appears to be important for the development of diseases associated with these risk factors. Most work has focused on miRNAs, and little is known about lncRNAs and their function in the development of diseases that are associated with tobacco and alcohol use.

A greater understanding of noncoding RNAs and their respective mechanisms might improve such outcomes and guide the development of novel diagnostic and treatment approaches. However, it is necessary to overcome limitations in integrating and validating the results due to the use of large-scale techniques.

## Figures and Tables

**Table 1:** List of microRNAs (miRNAs) altered by tobacco and alcohol exposition in distinct cancers.

Cancer	miRNA	Regulation *	Associated Risk Factor	Samples	Ref. ^#^
Lung	miR-486-5p	↓	Tobacco	Plasma	[63]
Lung	miR-625	↓	Tobacco	Serum and cell lines	[54]
Lung	miR-150	↓	Tobacco	Tumor tissues	[108]
Lung	miR-142-3p, miR-34c	↓	Tobacco	Tumor tissues	[109]
Lung	miR-126, miR-375, miR-486	↓	Tobacco	Sputum	[64]
Lung	miR-322, miR-326	↓	Tobacco	Mice	[68]
Lung	miR-29b-3p, miR-101-3p, miR-29a-3p, miR-193b-3p	↓	Tobacco	Mononuclear cells	[8]
Lung	miR-129-2-3p, let-7i-3p, miR-196a-3p, miR-154-5p	↓	Tobacco	Serum	[58]
Cholangio	miR-200	↓	Alcohol	Tumor tissues	[91]
Laryngeal **	miR-34c-5p	↓	Alcohol	Tumor tissues	[98]
Esophageal	miR-217	↓	Tobacco	Tumors and cell lines	[60]
Gastric	miR-143	↓	Tobacco	Tumor tissues	[70]
Lung	miR-7-5p, miR-502-3p, miR-200c-5p, miR-1301	↑	Tobacco	Serum	[58]
Lung	miR-23a, miR-181c, miR-192, miR-194, miR-208, miR-337-5p, miR-338-3p, miR-502-5p, miR-542-3p, miR-628-5p, miR-672	↑	Tobacco	Mucosal brushings	[66]
Lung	miR-21, miR-210	↑	Tobacco	Plasma	[63]
Lung	miR-100	↑	Tobacco	Tumor tissues	[59]
Lung	miR-224, miR-375, miR-452	↑	Tobacco	Tumor tissues	[109]
Lung	miR-20a, miR-223, miR-21, miR-145	↑	Tobacco	Plasma	[12]
Lung	miR-21, miR-31, miR-182, miR-200b, miR-205, miR-210, miR-708	↑	Tobacco	Sputum	[64]
Lung	miR-576-3p, miR-19b-3p, miR-766-3p	↑	Tobacco	Mononuclear cells	[8]
Lung	miR-21	↑	Tobacco	Serum and cell lines	[13]
Gastric	miR-21	↑	Tobacco	Brush cytology	[70]
Oral	miR-637	↑	Tobacco	Tumor tissues	[71]
Liver	miR-34	↑	Alcohol	Mice and cell lines	[30]
Liver	miR-21	↑	Alcohol	Mice and cell lines	[9]
Oral	miR-34	↑	Alcohol	Tumor tissues	[97]
Head and Neck	miR-30a-5p, miR-934, miR-3164, miR-3178	↑	Alcohol	Tumor tissues	[96]
Gastric	miR-203, miR-205, miR-223	↑	Alcohol	Tumor tissues	[70]

**Legends:** * Regulation: Green arrows indicate miRNA downregulation; Red arrows indicate miRNA upregulation; ** Premalignant lesion; Ref. ^#^: References; All miRNAs reported in this table have been altered by exposure to alcohol or tobacco.

**Table 2:** List of miRNAs altered by tobacco and alcohol exposure in other diseases.

Disease	miRNA	Regulation *	Associated Risk Factor	Samples	Ref. ^#^
**Cardiovascular Diseases**					
Myocardial Fibrosis	miR-133	↓	Tobacco	Cell line	[51]
Abdominal aortic aneurysms	miR-21	↑	Tobacco	Tissue, mice, cell line	[52]
Intestinal barrier dysfunction	miR-212	↑	Alcohol	Mice and cell line	[106]
**Respiratory diseases**					
COPD	miR-199a-5p	↓	Tobacco	Mononuclear cells	[78]
COPD	miR-146b-3p, miR-150, miR-210	↓	Tobacco	Bronchoalveolar lavage	[79]
COPD	miR-708, miR-200a, miR-210, miR-187, miR-149, miR-429, miR-146b-3p, miR-200c	↓	Tobacco	Alveolar macrophages	[34]
COPD	let-7, miR-125	↓	Tobacco	Sputum	[75]
COPD	miR-106b, miR-29b, miR-483-5p, miR-152, miR-629, miR 26b, miR-101, miR-106b, miR-532-5p, miR-133b	↓	Tobacco	Plasma	[82]
COPD	miR-7	↑	Tobacco	Cell line	[76]
COPD	miR-203	↑	Tobacco	Blood	[83]
COPD	miR-146a	↑	Tobacco	Cell line	[80]
**Liver diseases**					
Alcoholic liver diseases	miR-199-3p, miR-214, miR-93, miR-146a, miR-191, let-7b	↓	Alcohol	Tumor tissues	[103]
Steatosis	miR-217	↑	Alcohol	Mice and cell line	[105]
Alcoholic hepatitis	miR-27a	↑	Alcohol	Cell line and plasma	[101]
Alcoholic hepatitis	miR-192, miR-122, miR-30A, miR-744, miR-1246, miR-30, miR-130a	↑	Alcohol	Human plasma and mice	[102]
Hepatitis C Virus	miR-122	↑	Alcohol	Cell line	[92]
Alcoholic liver diseases	miR-122, miR-155, miR-146a	↑	Alcohol	Plasma and serum of mice	[93]
Alcoholic liver diseases	miR-570, miR-122, miR-34b, miR-29c, miR-922, miR-185	↑	Alcohol	Tumor tissues	[100]
Alcoholic liver diseases	miR-129, miR-490, miR-21, miR-503, miR-183, miR-185	↑	Alcohol	Tumor tissues	[103]

**Legends:** * Regulation: Green arrows indicate miRNA downregulation; Red arrows indicate miRNA upregulation; COPD: Chronic obstructive pulmonary disease; Ref. ^#^: References; All miRNAs reported in this table have been altered by exposure to alcohol or tobacco.
